# Targeted therapies to improve CFTR function in cystic fibrosis

**DOI:** 10.1186/s13073-015-0223-6

**Published:** 2015-09-24

**Authors:** Malcolm Brodlie, Iram J. Haq, Katie Roberts, J. Stuart Elborn

**Affiliations:** Institute of Cellular Medicine, Newcastle University and Department of Paediatric Respiratory Medicine, Great North Children’s Hospital, Queen Victoria Road, Newcastle, NE1 4LP UK; Department of Paediatric Respiratory Medicine, Great North Children’s Hospital, Queen Victoria Road, Newcastle, NE1 4LP UK; Centre for Infection and Immunity, Queen’s University Belfast, Health Sciences Building, 97 Lisburn Road, Belfast, BT9 7BL UK

## Abstract

Cystic fibrosis is the most common genetically determined, life-limiting disorder in populations of European ancestry. The genetic basis of cystic fibrosis is well established to be mutations in the cystic fibrosis transmembrane conductance regulator (*CFTR*) gene that codes for an apical membrane chloride channel principally expressed by epithelial cells. Conventional approaches to cystic fibrosis care involve a heavy daily burden of supportive treatments to combat lung infection, help clear airway secretions and maintain nutritional status. In 2012, a new era of precision medicine in cystic fibrosis therapeutics began with the licensing of a small molecule, ivacaftor, which successfully targets the underlying defect and improves CFTR function in a subgroup of patients in a genotype-specific manner. Here, we review the three main targeted approaches that have been adopted to improve CFTR function: potentiators, which recover the function of CFTR at the apical surface of epithelial cells that is disrupted in class III and IV genetic mutations; correctors, which improve intracellular processing of CFTR, increasing surface expression, in class II mutations; and production correctors or read-through agents, which promote transcription of *CFTR* in class I mutations. The further development of such approaches offers great promise for future therapeutic strategies in cystic fibrosis.

## Targeted therapies in respiratory medicine — cystic fibrosis as a paradigm

Targeted therapies have evolved in medicine following advances in molecular technology and the successful mapping of the human genome. Such treatments are well recognized in oncology, where molecules required for tumor growth and spread are specifically targeted to stop the malignant process or prevent tumor progression [1, 2].

These therapies have been driven by the concepts of precision and stratified medicine, whereby molecular biomarkers can be used to select specific approaches for individuals or groups of individuals, respectively, enabling the production of highly effective and precise treatments [3]. Some of the advantages of targeted therapies include the ability to identify treatment responders, tailor treatment to an individual’s genetic profile, and avoid unwanted side effects [4]. This approach is in direct contrast to most drugs currently used in medical practice, which are used to treat large populations with the same broad disease label but with marked heterogeneity in response to treatment.

Recent advances in genome-wide association studies and an increased understanding of the genetic basis of complex diseases have enabled the concept of targeted therapies to be investigated in other areas, such as respiratory medicine. However, there are few examples of targeted therapies in this field outside of oncological problems, as most lung diseases are complex and polygenic. Therefore, developing strategies for specific molecular abnormalities in these conditions is challenging. An exception, however, is cystic fibrosis, in which the underlying genetic defect is well defined and lies within the *CFTR* gene [5]. The use of ivacaftor, a potentiator of CFTR function, has become a successful reality since 2012 as a targeted therapy for patients with cystic fibrosis caused by specific genotypes, and represents a powerful example of precision medicine [6]. Furthermore, the combination of a potentiator and a corrector (ivacaftor and lumacaftor) received US Food and Drug Administration (FDA) approval in 2015 for use in people with cystic fibrosis caused by the most common *CFTR* mutation, Phe508del [7, 8].

In this review we discuss the clinical and genetic basis of cystic fibrosis, the development of treatments targeted at specific classes of *CFTR* mutation to address the basic defects and improve CFTR function, and the advent of precision medicine in cystic fibrosis as a paradigm for other respiratory diseases.

## Genetic causes, clinical manifestations and patient care

**Fig. 1:**
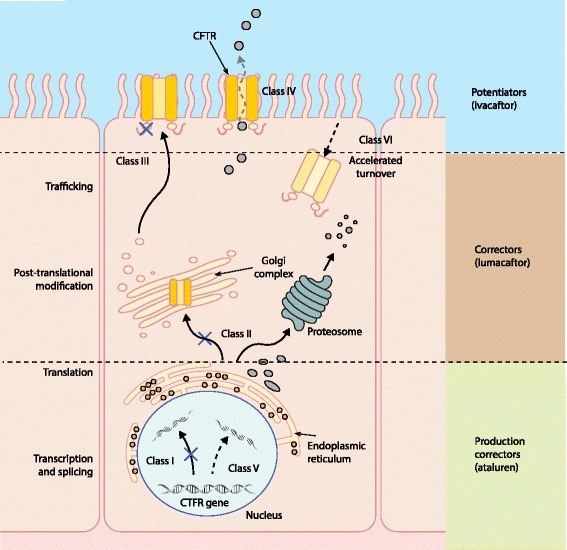
The different classes of *CFTR* gene mutations and the mechanisms of action of CFTR potentiators (such as ivacaftor), correctors (such as lumacaftor) and production correctors (such as ataluren). *CFTR* gene mutations are categorized into six classes. Mutation classes I, II, V and VI result in an absence or reduced quantity of CFTR protein at the cel membrane, whereas mutation classes III and IV influence the function or activity of CFTR at the cell membrane. Potentiators increase the function of CFTR channels expressed at the apical surface of epithelial cells; for example, ivacaftor increases the probability of Gly551Asp-CFTR channel opening. Correctors improve the intracellular processing and delivery of mutant CFTR protein, allowing more to reach the cell surface; for example, lumacaftor in Phe508del-CFTR. Production correctors (read-through agents) promote the read-through of premature termination codons in mRNA, generating more production of CFTR protein; for example, ataluren in class I CFTR mutations

Cystic fibrosis is the most common autosomal recessive life-limiting disorder [9]. It is particularly prevalent in northwestern European populations, with an incidence of around 1 in 2500 individuals, but it occurs in all populations worldwide. The *CFTR* gene was cloned 26 years ago and it encodes a chloride channel that is primarily expressed in epithelial cells [5]. Almost 2000 disease-causing mutations have been identified in people with cystic fibrosis to date, but a much smaller number of mutations account for the vast majority of cases [9, 10]. *CFTR* mutations may be classified into six different categories based on the mechanisms that are affected: CFTR synthesis, trafficking or function (Fig. 1 and Table 1).

**Table 1:** Summary of different classes of *CFTR* mutations

Mutation class	Nature of defect	Functional consequence	Example	Therapeutic strategy
I	CFTR protein synthesis	Reduced CFTR protein expression	Gly542X	Production correctors (ataluren)
II	CFTR protein processing	Misfolded CFTR not transported to cell surface	Phe508del	Corrector plus potentiator (lumacaftor plus ivacaftor, VX-661 plus ivacaftor)
III	CFTR channel gating	Reduced/lack of CFTR channel opening	Gly551Asp	Potentiator (ivacaftor)
IV	CFTR channel conductance	Misshaped CFTR pore restricts Cl^−^ movement	Arg117His	Potentiator (ivacaftor)
V	Reduced CFTR protein production	Very low levels of CFTR protein	3849 + 10 kb C → T	No data available
VI	High CFTR protein turnover at cell surface	Functional but unstable CFTR protein at cell surface	120del23	No data available

*kb* kilobases

Class I mutations result from nonsense, frameshift, or mRNA splicing mutations leading to absent CFTR production; for example, Gly542X, a nonsense mutation caused by a premature termination codon (PTC), results in an early translational defect and a truncated CFTR protein. Class II mutations, including Phe508del, are caused by defective CFTR processing. Although CFTR is correctly synthesized, missense and in-frame deletion mutations disrupt CFTR folding and trafficking to the cell surface. Class III mutations result in expression of CFTR at the cell membrane but channel gating is defective and results in impaired chloride transport function. For example, Gly551Asp, the most common class III mutation, eliminates the ability of ATP to increase the opening rate of CFTR [11]. Conductance defects are seen in class IV mutations, in which chloride transport is restricted owing to an abnormal CFTR channel pore. Class IV mutations, such as Arg117His, often result in a milder phenotype owing to partial CFTR function. Very low levels of CFTR are found in class V mutations, in which splicing defects lead to defective mRNA processing. Finally, class VI mutations are characterized by a functional but unstable CFTR, resulting in high CFTR turnover at the cell surface. To add further complexity to this classification system, certain mutations may lead to more than one class of functional defect, for example, Phe508del results in class II and III problems [12, 13]. Phe508del-CFTR is degraded at the level of the endoplasmic reticulum, with very little or zero mutant protein reaching the apical membrane of epithelial cells, which is typical of a class II *CFTR* mutation [14]. If Phe508del-CFTR is expressed at the apical membrane — for example, following monotherapy with a CFTR corrector such as lumacaftor — it has been demonstrated that the chloride channel has a reduced probability of being open, operating as a class III gating *CFTR* mutation [15, 16].

Cystic fibrosis is a multi-system disorder, but the vast majority of morbidity and mortality is associated with lung disease [9]. Lung disease in cystic fibrosis is characterized by neutrophilic inflammation, retention of mucoid secretions and chronic endobronchial infection with specific organisms, most notably *Pseudomonas aeruginosa.* Ultimately, this leads to progressive bronchiectasis and premature death in young adulthood. Other clinical problems in cystic fibrosis include malabsorption due to exocrine pancreatic insufficiency, diabetes and liver disease. The exact mechanism as to how a defective chloride channel causes such extensive lung problems is not fully understood and several hypotheses have been proposed. These include effects on the airway surface liquid (ASL; the thin layer of liquid that sits above the apical membrane of airway epithelial cells in which cilia beat) [17], compromised mucociliary clearance [18], alterations in ASL pH [19], defective innate immunity against pathogens [19], and pro-inflammatory responses in airway epithelial cells [20–22].

The development and implementation of various symptomatic treatments since cystic fibrosis was first recognized has greatly improved survival. In the 1970s and 1980s pancreatic enzyme replacement therapy and improved nutritional management were introduced [23]. In terms of lung disease, removal of mucus and secretions with chest physiotherapy [24] and more advanced lung clearance devices and techniques, along with the more recent use of inhaled drugs such as dornase alpha (which helps cleave DNA in the airway from necrotic neutrophils, reducing the tenacity of secretions) [25] and hypertonic saline (which works osmotically by increasing the hydration of mucus and ASL), have also been beneficial [26]. Furthermore, antimicrobial therapies targeting acute and chronic infection in the form of oral, intravenous and inhaled drugs are critical parts of modern cystic fibrosis treatment [27]. The development of specialist centers delivering expert multidisciplinary care in a coordinated fashion has also been crucial in improving outcomes [28]. At present, the median life expectancy of patients with cystic fibrosis has increased to nearly 40 years, with projected survival for newborn infants with cystic fibrosis born today beyond the fifth decade of life [29]. Genetic testing is a normal part of the diagnostic process, along with measurement of sweat chloride levels as a measure of CFTR function [30].

The treatments described above are all aimed at managing the downstream consequences of defective CFTR function and improving symptoms rather than tackling the underlying defect. Furthermore, these therapies represent a significant burden of care, involving physiotherapy multiple times a day along with numerous tablets and nebulizers. This burden is a particular problem in a lifelong condition such as cystic fibrosis, and adherence to treatment is a challenge even for the most dedicated of individuals [31].

## CFTR dysfunction as the underlying defect and potential therapeutic target

The relationship between the severity of disease in an individual and sweat chloride concentration as a readout of the level of CFTR function is complex. However, patients with mutations associated with lower levels of sweat chloride — for example, in the intermediate range — generally have improved survival, slower decline in lung function and a less severe overall phenotype [32]. This clinical observation confirms the logic of targeting CFTR to treat the fundamental defect in cystic fibrosis and makes it a highly attractive strategy [5].

Gene therapy to introduce the wild-type *CFTR* gene into airway epithelial cells in patients with cystic fibrosis so that they express functional CFTR has an obvious and elegant rationale. A large amount of careful and methodical research has been performed in this field over several decades [33]. The UK Cystic Fibrosis Gene Therapy Consortium recently published the results of a phase IIb trial of *CFTR* gene therapy delivered monthly for a year by nebulizer using a non-viral liposomal vector [34]. This has provided proof-of-concept that liposomal *CFTR* gene therapy is well tolerated and can provide clinical benefit in terms of lung function to patients with a broad range of *CFTR* genotypes. Interestingly, responses to gene therapy appeared to be heterogeneous and a greater treatment effect was observed in participants with lower baseline forced expiratory volume in one second (FEV_1_). It is very likely that further human studies will follow in the next few years to optimize dosing and to determine in which patient groups this approach might be most useful [34]. In the future, gene editing or correction in stem cells may become another genetic approach for cystic fibrosis therapy, a strategy that was recently demonstrated in 2013 in an ex vivo intestinal organoid model [35].

An alternative method to address the fundamental defect in cystic fibrosis is the development of genotype-specific small-molecule drugs that modulate CFTR function. Three main approaches have been adopted (Fig. 1). First, *potentiators* increase the function of CFTR channels expressed at the apical surface of epithelial cells and are used in class III or IV *CFTR* mutations, in which CFTR reaches the surface of cells but is dysfunctional. Second, *correctors* improve the intracellular processing and delivery of mutant CFTR protein in class II *CFTR* mutations, allowing more protein to reach the cell surface. Last, *production correctors* or *read-through agents* promote the read-through of PTCs in mRNA, allowing more production of the CFTR protein in class I *CFTR* mutations.

The simplest and most successful approach to date has been using small molecules to potentiate CFTR function in specific class III gating mutations in which CFTR is present at the cell membrane but is dysfunctional (Tables 1 and 2 and Fig. 1). The most frequently occurring class III mutation is Gly551Asp, which accounts for around 5 % of all mutant *CFTR* alleles in the population [11]. Gly551Asp is a missense mutation, in which the amino acid glycine is substituted for aspartate at position 551 in the nucleotide-binding domain-1 of the gene. Although the protein is present at the cell surface, the channel fails to open in response to ATP, resulting in defective chloride channel transport. A CFTR potentiator — such as ivacaftor — that specifically targets this gating mutation can increase CFTR function at the cell surface and in intracellular organelles [36].

**Table 2:** Summary of clinical studies investigating the efficacy of ivacaftor in patients with cystic fibrosis and the Gly551Asp mutation

Study name and reference	Accurso et al. 2010 [42]	STRIVE: Ramsey et al. 2011 [6]	ENVISION: Davies et al. 2013 [43]	Davies et al. 2013 [44]	Barry et al. 2014 [45]	
Type of study	Phase II RCT	Phase III RCT	Phase III RCT	Phase III RCT	Case–control study	
Number of participants	*n* = 39	*n* = 161	*n* = 52	*n* = 21	*n* = 56	
	Ivacaftor: 31; placebo: 8	Ivacaftor: 83; placebo: 78	Ivacaftor: 26; placebo: 22		Ivacaftor: 21; placebo: 35	
Duration	28 days	48 weeks	48 weeks	29 days	9 months	
Inclusion criteria	≥18 years	≥12 years	6–11 years	≥6 years	≥18 years	
	≥1 Gly551Asp allele	≥1 Gly551Asp allele	≥1 Gly551AspP allele	≥1 Gly551Asp allele	≥1 Gly551Asp allele	
	FEV_1_ > 40 %	FEV_1_ 40–90 %	FEV_1_ 40–105 %	FEV_1_ > 90 %	FEV1 < 40 %	
			Weight ≥15 kg	LCI >7.4	and/or actively listed for lung transplant	
				Weight ≥15 kg		
Outcome measure	Median change from baseline with 150 mg	Treatment effect	Treatment effect	Treatment effect	Changes within treated patients	Treated patients versus controls
Mean FEV_1_ (percentage predicted)	+8.7 (*P* = 0.008)	24 weeks: +10.6 (*P* < 0.001)	24 weeks: +12.5 (*P* < 0.001)	–	+4.2 (*P* = 0.0068)	+3.8 versus 0.6 (*P* = 0.009; median)
		48 weeks: +10.5 (P < 0.001)	48 weeks: +10 (*P* < 0.0006)			
Sweat chloride levels (mmol/L)	−59.5 (*P* = 0.008)	−47.9 (*P* < 0.001)	−54.3 (*P* < 0.001)	–	–	–
CFQ-R score (points)	+8.3 (*P* = 0.06)	+8.6 (*P* < 0.001)	+6.1 (*P* = 0.109)	–	–	–
Nasal potential difference (mV)	−3.5 (*P* = 0.02)	–	–	–	–	–
Weight (kg)	–	+2.7 (*P* < 0.001)	+2.8 (*P* < 0.001)	–	+1.8 (*P* = 0.0058; median)	+2.3 versus 0.6 (*P* = 0.25; median)
BMI	–	–	BMI-for-age *z*-score: 0.45 (*P* < 0.001)	–	+1.1 kg/m^2^ (*P* = 0.010; median)	+0.84 versus 0.2 kg/m^2^ (*P* = 0.234; median)
Time on intravenous antibiotics (days per year)	–	–	–	–	−36 (*P* = 0.0016; median)	−36 versus +10 (*P* = 0.0003; median)
Pulmonary exacerbations	–	55 % risk reduction	No significant difference	–	–	–
		(0.455 hazard ratio: *P* = 0.001)				
LCI	–	–	–	−2.16 (*P* < 0.0001)	–	–

*BMI* body mass index (the weight in kilograms divided by the square of the height in meters), *CFQ-R* revised Cystic Fibrosis Questionnaire, *FEV*_*1*_ percentage predicted forced expiratory volume in 1 second for age, sex and height, *LCI* lung clearance index, *RCT* randomized controlled trial

Importantly, the most common *CFTR* mutation by far is Phe508del, which is found on at least one chromosome in around 85 % of people with cystic fibrosis, and correcting the function of Phe508del-CFTR is much more challenging [14, 37]. The mutant Phe508del-CFTR protein is misfolded, which leads to intracellular degradation by the proteasome in the endoplasmic reticulum, with very little protein reaching the plasma membrane (class II mutation) [38]. However, *in vitro* cell culture work has demonstrated that if the temperature of cells is lowered or if they are treated pharmacologically, it is possible to bypass the degradation of Phe508del-CFTR and increase trafficking to the plasma membrane [39]. Once expressed at the membrane, Phe508del-CFTR then behaves as a gating (class III) mutation whose function could be potentiated in a similar fashion to Gly551Asp-CFTR [15, 16, 40]. Therefore, a dual CFTR corrector and potentiator approach is likely to be required to improve CFTR function in patients with the Phe508del mutation.

## The CFTR potentiatior ivacaftor

**Fig. 2:**
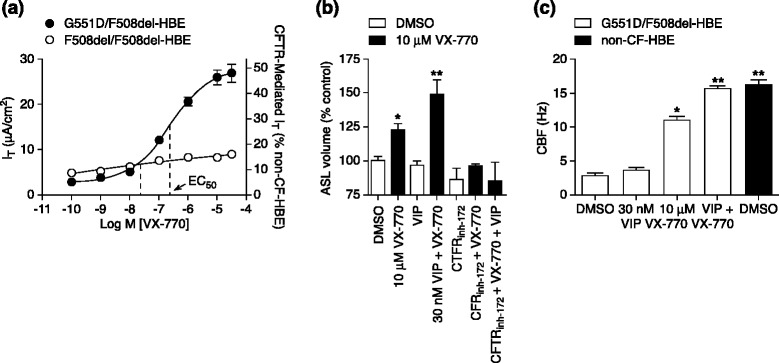
Summary of initial *in vitro* data on effects of ivacaftor (VX-770) on human bronchial epithelial cells (*HBEs*) expressing the Gly551Asp *CFTR* mutation. **a** Potentiation of CFTR-mediated chloride (Cl^−^) secretion following treatment with ivacaftor. Chamber techniques were used to record the transepithelial current (*I*_T_) resulting from CFTR-mediated Cl^−^ secretion. To isolate the CFTR-mediated *I*_T_, a basolateral-to-apical Cl^−^ gradient was established, 30 μM amiloride was added to block the epithelial sodium channel (ENaC), and 10 μM (maximal effective concentration; EC_99_) forskolin (*FSK*) was applied to activate the CFTR. The concentration–response curve for ivacaftor in the presence of FSK is shown for Gly551Asp/Phe508del HBEs isolated from the bronchi of a single individual (*filled circles*; *n* = 16) and Phe508del HBEs isolated from the bronchi of the three individuals who responded to ivacaftor (*open circles*; *n* = 7–24). Left *y*-axis shows *I*_T_ responses; right *y*-axis shows *I*_T_ normalized to the 10 μM FSK-stimulated *I*_T_ in non-cystic fibrosis (CF) HBEs (mean ± standard error of the mean). Note that the error bars for the Phe508del HBEs were smaller than the symbol. **b** Increased airway surface liquid (*ASL*) following treatment with ivacaftor. Mean (*n* = 3–9) ASL volume in the absence (*open bars*) or presence (*filled bars*) of 10 μM ivacaftor and in the presence of 30 nM vasoactive intestinal peptide (*VIP*) and/or 20 μM CFTR inhibitor-172 (*inh-172*). **c** Increased ciliary beat frequency (*CBF*) following treatment with ivacaftor. Mean (± standard error of the mean; *n* = 6) CBF for wild-type HBEs (*filled bars*) or Gly551Asp/Phe508del HBEs (*open bars*) after a 5-day treatment with DMSO, 30 nM VIP, 10 μM ivacaftor, or 30 nM VIP with 10 μM ivacaftor. *Single asterisk* indicates significantly different (*P* < 0.05) from vehicle control in Gly551Asp/Phe508del HBEs; *double asterisk* indicates significantly different (*P* < 0.05) from vehicle control and ivacaftor alone. *EC*_*50*_ half-maximum effective concentration. Reproduced with permission from Van Goor et al. [36]

Ivacaftor (VX-770) is a small-molecule drug that was identified via high-throughput screening involving a library of nearly 230,000 potential therapeutic compounds [41]. Further *in vitro* evaluation demonstrated that ivacaftor significantly augmented chloride transport and increased ASL height and cilia beat frequency in airway epithelial cells expressing Gly551Asp-*CFTR* mutation (Fig. 2) [36]. This watershed moment in cystic fibrosis therapeutics then led to fast-tracked clinical trials to investigate the efficacy of ivacaftor as an orally bioavailable drug in patients who had at least one Gly551Asp allele.

### Clinical trials of ivacaftor in patients with the Gly551Asp *CFTR* mutation

A phase II double-blinded placebo-controlled trial was performed to determine the safety, efficacy and adverse outcomes of different doses of ivacaftor and to ascertain any clinical improvements with treatment versus placebo (Table 2) [42]. Biomarkers of CFTR function, lung function and quality of life measures were also assessed [42].

Measures of CFTR function included nasal potential difference (as a marker of chloride conductance in the nasal epithelium) and sweat chloride concentration (as a readout of chloride reabsorption by CFTR in the sweat duct). Lung function was measured in terms of percentage of predicted FEV_1_. Quality of life was assessed using the revised Cystic Fibrosis Questionnaire (CFQ-R). Patients aged 18 years or older who had at least one Gly551Asp allele and a predicted FEV_1_ of at least 40 % were included in the study. The first stage of the study had a crossover design in which patients were randomly assigned to receive varying doses of ivacaftor (25 mg, 75 mg, 150 mg or placebo) every 12 hours over two 14-day periods with a washout time between these periods. The second stage was a parallel study, which involved new patients who were randomly assigned to receive varying doses of ivacaftor (150 mg, 250 mg or placebo) every 12 hours over 28 consecutive days.

Of 39 patients, 31 (79 %) received ivacaftor and 8 (21 %) received the placebo. In general, ivacaftor was well tolerated. The study found a partial improvement in nasal potential difference and significant improvements in sweat chloride concentrations in individuals who received ivacaftor. The median reduction in sweat chloride levels after 28 days in the 150 mg ivacaftor group was −59.5 mmol/L versus a gain of 5 mmol/L in the placebo group. Interestingly, although still significant, the reduction in sweat chloride levels was smaller in those who received 250 mg than in those who received 150 mg of the drug. There were no significant improvements in FEV_1_ in patients who received ivacaftor versus placebo. However, there were significant within-subject improvements in FEV_1_ from baseline with the 75 mg and 150 mg doses. Health-related quality of life was better with ivacaftor, but the changes were not significant. Although this study clearly demonstrated the benefits of ivacaftor in patients with cystic fibrosis, it was limited by its small sample size, which may have contributed towards the lack of significance found with some outcome measures.

Two larger phase III double-blinded randomized controlled trials (RCTs) were then performed to evaluate the efficacy of ivacaftor in patients with the Gly551Asp *CFTR* mutation (Table 2) [6, 43]. Participants in both studies were randomly assigned in a 1:1 fashion to receive either 150 mg of ivacaftor every 12 hours or placebo during a 48-week period. The STRIVE study was carried out in patients aged 12 years and over, and the ENVISION study looked at younger children aged 6–11 years. The primary outcome of both studies was percentage predicted FEV_1_ at 24 weeks, with secondary outcomes including FEV_1_ at 48 weeks, weight, sweat chloride levels, CFQ-R symptom score and safety profile. In the STRIVE study, time to first pulmonary exacerbation was also a secondary endpoint.

Both studies demonstrated a significant improvement in lung function with ivacaftor. In STRIVE, the effect of treatment on percentage predicted FEV_1_ at 24 weeks was 10.6 percentage points (*P* < 0.001) and at 48 weeks was 10.5 percentage points (*P* < 0.001) [6]. In the ENVISION study this effect was 12.5 percentage points (*P* < 0.0001) and 10 percentage points (*P* < 0.0006) at 24 and 48 weeks, respectively [43]. In both studies, significant improvements were noted as early as 15 days. In STRIVE, a 55 % risk reduction of pulmonary exacerbations was observed with ivacaftor therapy versus placebo at 48 weeks [6]. This effect was not seen in the ENVISION study, which was not powered to detect such a change; the number of pulmonary exacerbations was small and similar between the placebo (three events) and the ivacaftor (four events) groups [43]. This can be explained by the younger age and relatively milder disease seen in ENVISION participants compared to STRIVE, with mean baseline FEV_1_ values at 63 % and 84 % predicted, respectively.

In the STRIVE study, the time spent in hospital was significantly reduced in patients taking ivacaftor. Mean duration of hospitalization per patient was 3.9 days (±13.6) in ivacaftor patients versus 4.2 days (±8.7) in the placebo group. Ivacaftor also reduced the number of exacerbations requiring intravenous treatment by 26 % and hospitalization by 15 % [6].

These studies clearly demonstrated significant improvements in sweat chloride levels at 24 and 48 weeks. In STRIVE there was a reduction in sweat chloride levels of 48.7 mmol/L at 24 weeks compared with 0.8 mmol/L in the placebo group (treatment effect −47.9 mmol/L, *P* < 0.001) [6]. Interestingly, the mean sweat chloride concentrations at this stage were 47.8 mmol/L in the ivacaftor group versus 100 mmol/L in the placebo group. The rapid reduction in sweat chloride levels was also seen in the ENVISION study (treatment effect 54.3 mmol/L, *P* < 0.001) [43]. Reductions were sustained at 48 weeks in both studies.

Improvements in respiratory symptoms were seen in individuals taking ivacaftor in both studies. The CFQ-R score was significantly improved in the older patients (treatment effect of 8.6 points, *P* < 0.001) [6]. Although there was an improvement in patients taking ivacaftor in the ENVISION trial, the findings were not significant (treatment effect of 6.1 points, *P* = 0.109) [43]. Baseline CFQ-R was higher in younger patients, which suggests that they had relatively milder disease and symptoms. Parents and caregivers also completed a version of the CFQ-R, which, interestingly, did reveal significant improvements. This discrepancy perhaps reflects the challenges faced when completing and interpreting health-related questionnaires in children. Patients taking ivacaftor in both studies showed promising improvements in weight gain. At 48 weeks, the effect of treatment was 2.7 kg in STRIVE (*P* < 0.001) and 2.8 kg (*P* < 0.001) in ENVISION [6, 43].

The most common adverse symptoms were headache, nasal congestion, upper respiratory tract infection, rash and dizziness. These effects were similar in the ivacaftor and placebo groups and all participants were able to continue treatment [6, 43]. In general, there were fewer serious adverse events in people taking ivacaftor in both studies compared with those receiving placebo. These events included pulmonary exacerbations, productive cough, hemoptysis and hypoglycemia. One patient receiving ivacaftor in STRIVE discontinued treatment owing to raised hepatic enzyme levels [6]. This effect was not seen in ENVISION, and in both studies there were no clinically relevant abnormal laboratory results or physical examination findings attributable to ivacaftor [6, 43].

In a subsequent phase II crossover study involving patients aged over 6 years with mild impairment of lung function, treatment with ivacaftor for 28 days was associated with improvements in lung clearance index compared with placebo (Table 2) [44]. Lung clearance index is measured using multiple-breath washout and is a sensitive method to assess changes in lung function in patients with cystic fibrosis for whom spirometry is in the normal range [44].

The effects of ivacaftor have also been studied in patients with severe impairment of lung function with an FEV_1_ percent predicted to be ≤40 % (Table 2) [45]. Significant improvements in lung function, number of days of intravenous antibiotic therapy, weight and body mass index (BMI) were found among patients after treatment in a retrospective case–control study [45]. In particular, reductions in treatment requirements — for example, number of inpatient days for intravenous antibiotics (median days per year reduced from 23 to 0, *P* = 0.001) — were felt to be clinically significant and greater than in studies involving patients with less severe lung disease [45].

In summary, the studies described above have clearly shown that ivacaftor is associated with significant health improvements in patients with the Gly551Asp *CFTR* mutation, who account for 5 % of people with cystic fibrosis, and have highlighted the beneficial role of this drug as a targeted therapy [46].

### Clinical trials of ivacaftor in patients with other *CFTR* mutations

Gating mutations other than Gly551Asp account for around 1 % of all *CFTR* mutations; individually, many of these mutations are rare. In addition to Gly551Asp, there is also *in vitro* evidence that ivacaftor potentiates CFTR function in other class III *CFTR* mutations [36, 47]. Furthermore, ivacaftor also potentiates CFTR function *in vitro* in cells expressing CFTR with some residual function (class IV *CFTR* mutations) [48]. These mutations include the Arg117His *CFTR* missense mutation that causes mixed conductance (class IV) and gating (class III) abnormalities, which is responsible for around 2 % of *CFTR* mutations in northern European populations [49]. It was hypothesized, therefore, that ivacaftor might be of potential benefit to people with cystic fibrosis with other class III and IV mutations, and clinical studies were undertaken to investigate its clinical efficacy in those scenarios (Table 3).

**Table 3:** Summary of clinical studies investigating the efficacy of ivacaftor in patients with cystic fibrosis mutations other than Gly551Asp

Study name and reference	Flume et al. 2012 [54]	KONNECTION: De Boeck et al. 2014 [51]	KONDUCT: Moss et al. 2015 [53]
Type of study	Phase II RCT with open label extension	Phase III randomized crossover trial with open label extension	Phase III RCT
Number of participants	*n* = 104	*n* = 39	*n* = 69
			Ivacaftor 34; placebo 35
Duration	16 weeks (96-week extension)	24 weeks (total)	24 weeks
		8 weeks placebo/ivacaftor	
		8 weeks ivacaftor/placebo	
		12 weeks ivacaftor	
Inclusion criteria	≥12 years	≥6 years	≥6 years
	Phe508del homozygous	>1 non-Gly551Asp gating mutation	>1 Arg117His mutation
	FEV_1_ > 40 %	FEV_1_ > 40 %	FEV_1_ > 40–90 % (>12 years)
			FEV_1_ > 40–105 % (6–11 years)
			Weight >15 kg
Outcome measure	Treatment effect	Treatment effect after 8 weeks	Treatment effect
Mean FEV_1_ (percentage predicted)	+1.7 (*P* = 0.15)	+10.7 (*P* < 0.0001)	All ages: +2.1 (*P* = 0.2)
			>18 years: +5 (*P* = 0.01)
			6–11 years: −6.3 (*P* = 0.03)
Sweat chloride levels (mmol/L)	−2.9 (*P* = 0.04)	−49.2 (*P* < 0.0001)	−24 (*P* < 0.0001)
CFQ-R score (points)	No significant differences	+9.6 (*P* = 0.0004)	+8.4 (*P* = 0.009)
Weight (kg)	No significant differences	–	–
BMI	No significant differences	BMI-for-age *z*-score 0.28 (*P* = 0.001)	–

*BMI* body mass index (the weight in kilograms divided by the square of the height in meters), *CFQ-R* revised Cystic Fibrosis Questionnaire, *FEV*_*1*_ percentage predicted forced expiratory volume in 1 second for age, sex and height, *RCT* randomized controlled trial

As mentioned earlier, the Phe508del *CFTR* mutation is much more common and is present in around 85 % of people with cystic fibrosis. The biology of the Phe508del-CFTR protein poses a much greater challenge to improve function. However, *in vitro* studies have shown that the defective Phe508del-CFTR protein has a limited response to CFTR potentiation [50], indicating that ivacaftor could also potentially benefit patients with this mutation type. This hypothesis was also tested in clinical studies, described in detail below.

#### Non-Gly551Asp *CFTR* gating mutations

The KONNECTION study was a two-part randomized international multicenter study designed to investigate the safety and efficacy of ivacaftor in patients with cystic fibrosis over the age of 6 years with a non-Gly551Asp *CFTR* gating mutation (Table 3) [51]. Part 1 was an 8-week blinded placebo-controlled crossover study with a 4–8-week washout period, and part 2 was an open-label extension period of 24 weeks. The primary outcome of each part of the study was percentage predicted FEV_1_, with secondary outcomes including BMI, sweat chloride levels and quality of life assessed with the respiratory domain of the CFQ-R. There was a statistically significant increase in FEV_1_ of 7.5 percentage points in the ivacaftor group in part 1 and 13.5 percentage points in part 2. BMI and sweat chloride levels decreased and quality of life improved with ivacaftor therapy in each part of the study. Subgroup analysis confirmed these findings for individual genotypes, with the exception of patients with the Gly970Arg mutation, in which there was a substantially less pronounced reduction in sweat chloride levels. As in the Gly551Asp studies, ivacaftor was generally well tolerated, with similar adverse events in the placebo and treatment groups [51].

#### Arg117His *CFTR* mutation

The phenotype associated with the Arg117His mutation is variable, depending on the other *CFTR* mutation present and the presence of a polypyrimidine variant in the intron 8 acceptor splice site; the mutation is often associated with less severe clinical problems [52].

The KONDUCT study was a phase III randomized controlled 24-week trial of ivacaftor versus placebo in people aged ≥6 years with an Arg117His *CFTR* mutation (Table 3) [53]. The primary outcome was change in percentage predicted FEV_1_, with secondary outcomes including changes in BMI, sweat chloride levels and the respiratory domain of CFQ-R, as well as safety. A total of 69 patients were recruited in this multicenter study.

The primary outcome was not statistically significant, with an increase in FEV_1_ in the treatment group of 2.1 percentage points compared with placebo (*P* = 0.2). Secondary outcome differences in CFQ-R scores (+8.4, *P* = 0.009) and sweat chloride levels (−24 mmol/L, *P* < 0.001) were statistically significant. The change in BMI in the treatment group was not statistically significant (+0.26 kg/m^2^, *P* = 0.78). No new safety concerns were identified [53].

A pre-specified subgroup analysis for the participants aged over 18 years (*n* = 50), who had a substantially lower average baseline percentage predicted FEV_1_ of around 65 % compared with around 95 % in those aged less than 18 years, showed a statistically significant increase in the primary outcome of FEV_1_ (+5 percentage points, *P* = 0.01) in the treatment group. Subgroup analysis of the participants aged less than 18 years, who had a higher baseline FEV_1_ of 95.8 %, showed an actual decline in lung function in the treatment group (−6.3 percentage points, *P* = 0.03). An open-label extension study was performed after washout for 65 of the original trial participants. Pooled results of the extension study showed an improvement in percentage predicted FEV_1_ of +5.1 percentage points at week 12 compared with post-washout baseline values (*P* < 0.0001) [53]. Overall, these results suggest a potential benefit of ivacaftor in patients with the Arg117His *CFTR* mutation and more advanced lung disease.

#### Phe508del *CFTR* mutation

A phase II study was performed in patients who were homozygous for Phe508del [54]. A total of 140 clinically stable patients aged ≥12 years were randomized in a blinded fashion to receive ivacaftor or placebo for 16 weeks, which was followed by an open-label extension period for patients who had demonstrated a pre-specified clinical response in the first part of the study. The primary efficacy outcome was change in percentage predicted FEV_1_ in the randomized part of the study, with safety also assessed, along with secondary outcomes including sweat chloride concentration, weight and CFQ-R scores.

There was no statistically significant increase in percentage predicted FEV_1_ in the treatment group versus the placebo group in the first part of the study (+1.7 percentage points, *P* = 0.15), with a very small reduction in sweat chloride levels (−2.9 mmol/L, *P* = 0.04). These changes were not sustained in the open-label phase. The safety profile of ivacaftor was similar to that of placebo with no new concerns raised. The conclusion of this study was that ivacaftor as a CFTR potentiator alone is not an effective therapeutic strategy for patients with cystic fibrosis who are homozygous for the Phe508del mutation [54]. This finding is perhaps not surprising owing to the very low or no expression of Phe508del-CFTR at the apical surface of epithelial cells in this patient group (Fig. 1 and Table 1) [55].

### Clinical use of ivacaftor in the post-approval setting

Ivacaftor was designated as an orphan medicine by the European Union in 2008, and since 2012 has been commissioned for use in the United Kingdom in patients aged 6 years or older with at least one Gly551Asp allele. It is also prescribed in most of the rest of Europe, the United States and other countries with well-developed clinical services for cystic fibrosis. Ivacaftor is an expensive drug and, for example, in the United Kingdom it is currently available under a Patient Access Scheme as a means to improve the cost effectiveness of treatment. Although such mutations are individually rare, in 2014 the European Union granted approval to ivacaftor for the treatment of people with one of eight other non-Gly551Asp gating *CFTR* mutations. In December 2014 the US FDA also approved the use of ivacaftor in patients with the Arg117His *CFTR* mutation.

Whether ivacaftor has definitive long-term benefits remains uncertain but preliminary data seem promising. Patients from the ENVISION and STRIVE studies were rolled over to an extended study to investigate the benefits of ivacaftor over a total period of 144 weeks, called PERSIST (Table 2) [56]. Both groups showed a sustained improvement in absolute percentage of predicted FEV_1_ and weight [56]. Furthermore, participants who received placebo in the original studies responded in a similar fashion to those who received ivacaftor originally [56]. During the extended study, the adverse event rate remained similar to previously and no new safety concerns were identified [56]. Improvements in lung function and nutrition have also been demonstrated in the post-approval setting in people with advanced cystic fibrosis-related lung disease [45, 57, 58].

Despite the exciting and groundbreaking benefits associated with ivacaftor, over 90 % of patients with cystic fibrosis will not benefit from monotherapy with this agent. Developing approaches that are targeted at other common *CFTR* mutations is therefore a clear priority.

## Other CFTR-targeted therapeutic approaches

### Ataluren (PTC124) as a production corrector or read-through agent

Ataluren is an orally bioavailable agent that has been trialed for use in class I *CFTR* mutations, which affect around 10 % of patients with cystic fibrosis [59]. These so-called ‘nonsense’ mutations involve a PTC. The PTC results in the interruption of ribosomal translation, resulting in a shortened, unstable and non-functional CFTR protein [60, 61]. Thought to be similar in function to aminoglycosides, ataluren enables ribosomes to skip this PTC, acting as a production corrector or ‘read-through agent’, leading to the formation of functional protein (Fig. 1). The actual mechanism of action of ataluren has been challenged, however, and the drug has been demonstrated to have some off-target effects on a reporter assay using firefly luciferase activity in drug development [62]. Ataluren has also been trialed in Duchenne muscular dystrophy caused by nonsense mutations and has been granted conditional marketing authorization for this indication in the European Union [63].

A phase II prospective clinical trial of ataluren was performed in adult patients with cystic fibrosis and a class I *CFTR* mutation; the trial included 23 participants in the first cycle and 21 in the second (Table 4) [64]. Each cycle varied in the dose of ataluren used but involved 14 days of treatment, with assessment of CFTR-mediated chloride transport by measurements of the transepithelial nasal potential difference. There was no placebo group. Oral treatment with ataluren was associated with an improvement in electrophysiological profile in the majority of patients. In 13 patients in cycle 1 and 9 patients in cycle 2, total chloride transport entered the normal range. Ataluren was generally well tolerated by the study participants. A further 19 patients entered a follow-on study, in which they received varying doses of ataluren for another 12 weeks. The results of this study showed ongoing improvement in CFTR function as assessed by evaluation of the nasal potential difference and raised no new safety issues [65].

**Table 4:** Summary of clinical studies investigating the efficacy of ataluren in patients with nonsense cystic fibrosis mutations

Study name and reference	Kerem et al. 2008 [64]	Sermet-Gaudelus et al. 2010 [46]	Wilschanski et al. 2011 [65]	Kerem et al. 2014 [66]
Type of study	Phase II randomized crossover trial	Phase II randomized crossover trial	Extension of trial by Kerem et al. 2008 [45]	Phase III RCT
Number of participants	*n* = 23	*n* = 30	*n* = 19	*n* = 238
Duration	Cycle 1: 16 mg/kg/day for 14 days; no treatment for 14 days	2 × 28 days	12 weeks	48 weeks
	Cycle 2: 40 mg/kg/day for 14 days; no treatment for 14 days	Cycle 1: 16 mg/kg/day for 14 days; no treatment for 14 days Cycle 2: 40 mg/kg/day for 14 days; no treatment for 14 days	Group 1: 16 mg/kg/day Group 2 : 40 mg/kg/day	
Inclusion criteria	≥18 years	6–18 years	≥18 years	≥6 years
	2 disease mutations, >1 nonsense	2 disease mutations, >1 nonsense	2 disease mutations, >1 nonsense	Nonsense mutations
	Sweat chloride >40 mmol/L	Sweat chloride >40 mmol/L	Sweat chloride >40 mmol/L	Sweat chloride >40 mmol/L
	Abnormal nasal potential difference	Abnormal nasal potential difference	Abnormal nasal potential difference	Abnormal nasal potential difference
	FEV_1_ > 40 %	FEV_1_ > 40 %	FEV_1_ > 40 %	FEV_1_ 40–90 %
	O_2_ saturation ≥92 % room air	O_2_ saturation ≥92 % room air	O_2_ saturation ≥92 % room air	O_2_ saturation ≥92 % room air
		Weight ≥25 kg		Weight ≥16 kg
Outcome measure	Treatment effect	Treatment effect	Treatment effect	Treatment effect
Mean FEV_1_ (percentage predicted)	Small increase (*P* = 0.037)	No significant difference	No significant difference	+3 % (*P* = 0.12)
Sweat chloride levels (mmol/L)	No significant difference	–	–	–
Chloride transport	Cycle 1: −7.1 (*P* < 0.0001)	Cycle 1: −4.6 mV (*P* = 0.037)	Group 1: −6.8 (*P* < 0.004)	–
	Cycle 2: −3.7 (*P* = 0.032)	Cycle 2: −3.9 mV (*P* = 0.046)	Group 2: −3.4 (*P* = 0.025)	
Nasal potential difference (mV)	(Change in basal nasal potential difference)	–	–	–
	Cycle 1: +3.3 (*P* = 0.04)			
	Cycle 2: +3.1 (*P* = 0.13)			
Weight (kg)	+0.6 kg (*P* < 0.0001)	No significant difference	–	–
Pulmonary exacerbations	–	–	–	Rate ratio 0.77 (*P* = 0.0992)

*FEV*_*1*_ percentage predicted forced expiratory volume in 1 second for age, sex and height, *RCT* randomized controlled trial

Another study of ataluren was performed in children and young people with cystic fibrosis aged between 6 and 18 years with a class I *CFTR* mutation [59]. Similar to the adult study described above, there were two 14-day cycles of oral treatment, with varying doses in each cycle, and the primary outcome was nasal potential difference. In around half of the participants an electrophysiological response was demonstrated with ataluren treatment and the agent was generally well tolerated. Evidence was also demonstrated of increased CFTR expression by nasal epithelial cells after treatment [59].

These results led to a phase III RCT, the results of which were published in 2014 [66]. In this study, 238 patients were recruited and randomized to receive placebo or ataluren for 48 weeks. The primary outcome was change in percentage predicted FEV_1_, with the number of pulmonary exacerbations as a secondary outcome. There was no statistically significant difference between ataluren treatment and placebo for the primary outcome (−2.5 versus −5.5 percentage points for FEV_1_, *P* = 0.12). The secondary outcome was also not statistically significantly different between groups. A post hoc analysis was performed in patients not taking inhaled tobramycin regularly. The analysis showed that this group had a significant increase in FEV_1_ of around 5 % with ataluren treatment compared with placebo (−0.7 % versus −6.4 %, *P* = 0.0082), along with a reduction in the number of pulmonary exacerbations [66]. The authors concluded that the drug may be of benefit to those not receiving inhaled tobramycin treatment, but it should be noted that, to date, no phase III trial of ataluren in cystic fibrosis has met its endpoints [66]. It has been hypothesized that tobramycin interferes with the mechanism of action of ataluren [66], and a further phase III study is underway to investigate the efficacy of ataluren in patients not receiving this antibiotic (ClinicalTrials.gov identifier NCT02139306) [67].

### The CFTR corrector lumacaftor

Lumacaftor (VX-809) is an example of a CFTR corrector. *In vitro* studies have shown that lumacaftor improves CFTR processing and chloride secretion in bronchial epithelial cells derived from people with cystic fibrosis homozygous for Phe508del [40]. A subsequent RCT investigated the safety and effect on CFTR function of lumacaftor monotherapy in Phe508del homozygotes [68]. This trial was a randomized placebo-controlled study involving treatment with varying doses of the drug for 28 days. The primary endpoints were the safety and tolerability of lumacaftor, with secondary outcomes including measures of CFTR function (sweat chloride levels and nasal potential difference), percentage predicted FEV_1_ and CFQ-R score. Treatment with lumacaftor had a similar safety and adverse event profile to treatment with placebo and no major safety concerns were raised. Modest but statistically significant dose-dependent improvements in sweat chloride levels were demonstrated with lumacaftor treatment. No changes with lumacaftor were demonstrated in participants who underwent nasal potential difference measurements. The study failed to find any significant improvements in lung function or CFQ-R outcomes associated with the drug. However, the study was not powered for these endpoints, and the authors comment that longer and larger trials would be required to assess these endpoints adequately [68].

### Combination therapies

As discussed earlier, the biology associated with the most common *CFTR* mutation, Phe508del, is particularly complex, leading principally to both class II and III defects [12, 14, 15]. This provides an explanation for the disappointing results associated with lumacaftor monotherapy in patients who are homozygous for the mutation [68] — if Phe508del-CFTR is expressed at the apical membrane, the chloride channel still has a (reduced) probability of being open [15, 39]. To add a further layer of complexity, Phe508del-CFTR also demonstrates reduced surface stability if it reaches the apical membrane [13].

#### Lumacaftor and ivacaftor

Conceptually, the combination of lumacaftor, to correct intracellular processing of CFTR, with ivacaftor, to potentiate the function of CFTR once it is trafficked to the plasma membrane, is highly attractive for patients with the Phe508del *CFTR* mutation [69].

The results of a complex phase II RCT investigating the effects of varying doses of lumacaftor in combination with ivacaftor versus placebo in adult Phe508del patients were published in 2014 (Table 5) [70]. The trial involved three successive cohorts of participants, with the results of earlier cohorts informing optimal dosing of lumacaftor for subsequent ones. The first cohort consisted of 64 Phe508del homozygous patients, and the second and third cohorts included 96 homozygotes and 28 compound heterozygotes. The primary outcomes were change in sweat chloride levels and safety, with secondary outcomes including percentage predicted FEV_1_. A modest yet statistically significant reduction in sweat chloride levels was demonstrated in treatment groups compared with placebo groups, as well as an increase of 5.6 percentage points in predicted FEV_1_ in the highest lumacaftor dose group. There was no significant change in FEV_1_ in the compound heterozygous subgroup. The frequency and nature of adverse events were similar between treatment and placebo groups, with no new concerns raised during the study.

**Table 5:** Summary of clinical studies investigating the efficacy of lumacaftor and ivacaftor in patients with Phe508del mutations

Study name and reference	Boyle et al. 2014 [70]	TRAFFIC and TRANSPORT: Wainwright et al. 2015 [8]
Type of study	Phase II RCT	Phase III RCT
Number of participants and study design	Cohort 1:	Cohort 1:
	64 homozygotes	*n* = 368
	Lumacaftor 200 mg/day for 14 days	Lumacaftor 600 mg/day plus ivacaftor 250 mg every 12 h
	Followed by:	Cohort 2:
	Ivacaftor 150 mg/250 mg every 12 h for 7 days	*n* = 369
	OR	Lumacaftor 400 mg every 12 h plus ivacaftor 250 mg every 12 h
	Placebo for 21 days	Cohort 3:
	Cohorts 2 and 3:	*n* = 371
	96 homozygotes	Placabo plus placebo
	28 compound heterozygotes	
	Cohort 2:	
	Lumacaftor 200 mg, 400 mg, 600 mg/day for 56 days	
	Cohort 3:	
	Lumacaftor 400 mg every 12 h for 56 days	
	Followed by:	
	Ivacaftor 250 mg every 12 h after 28 days	
	OR	
	Placebo for 56 days	
Duration	Cohort 1: 21 days	24 weeks
	Cohorts 2 and 3: 56 days	
Inclusion criteria	≥18 years	≥12 years
	>1 Phe508del allele	Phe508del homozygous
	FEV_1_ > 40 %	FEV_1_ 40–90 %
Outcome measure	Treatment effect	Pooled analysis of treatment effect in TRAFFIC and TRANSORT
Mean FEV_1_ (percentage predicted)	Cohort 2 with lumacaftor 600 mg/day: +5.6 (*P* = 0.013)	Cohort 1: +3.3 (*P* < 0.001)
	Cohort 3: no significant differences	Cohort 2: +2.8 (*P* < 0.001)
Sweat chloride levels (mmol/L)	Cohort 1 with 250 mg ivacaftor: −9.1 mmol/L (*P* < 0.001) Cohorts 2 and 3: no significant differences	–
CFQ-R score (points)	–	Cohort 1: 3.1 (*P* = 0.007)
		Cohort 2: 2.2 (*P* = 0.05)
BMI	–	Cohort 1: 0.28 (*P* < 0.001)
		Cohort 2: 0.24 (*P* < 0.001)
Pulmonary exacerbations	–	Cohort 1: rate ratio 0.7 (*P* = 0.001)
		Cohort 2: rate ratio 0.61 (*P* < 0.001)

*BMI* body mass index (the weight in kilograms divided by the square of the height in meters), *CFQ-R* revised Cystic Fibrosis Questionnaire, *FEV*_*1*_ percentage predicted forced expiratory volume in 1 second for age, sex and height, *RCT* randomized controlled trial

The results from this phase II study were sufficiently strong to support two phase III RCTs being undertaken in homozygous Phe508del patients to investigate the efficacy and safety of lumacaftor/ivacaftor combination therapy (TRAFFIC and TRANSPORT) [8]. The results of these studies were published in May 2015 [8]. Each study was randomized, double-blinded and placebo-controlled, and recruited patients aged ≥12 years who were homozygous for Phe508del. Participants were randomized to receive placebo or one of two different lumacaftor doses (400 mg twice daily or 600 mg once daily) and ivacaftor (250 mg twice daily). The primary outcomes were absolute change from baseline in percentage predicted FEV_1_, with secondary endpoints including relative change in percentage predicted FEV_1_, BMI, rate of pulmonary exacerbations and CFQ-R scores. Both TRAFFIC and TRANSPORT were large (559 and 563 patients recruited, respectively), multicenter studies that involved 24 weeks of treatment. The average baseline lung function of patients was around 60 % predicted FEV_1_.

There was a statistically significant increase in predicted FEV_1_ of between 2.6 and 4 percentage points in the treatment groups compared with placebo groups (*P* < 0.001). This difference was maintained in subgroup analyses stratified by age, percentage predicted FEV_1_ and *P. aeruginosa* infection status. In pooled analyses including data from both studies, the rate of pulmonary exacerbations was around a third lower in the treatment groups and BMI increased by approximately 1 % [8]. In terms of CFQ-R, a significant improvement in pooled analyses was only observed for the 600 mg once-daily lumacaftor treatment group. Seven patients in the treatment group experienced serious adverse events relating to deranged liver function, which were normalized with discontinuation of the study drug. Otherwise, safety profiles were similar among placebo and treatment groups. There was an increased rate of discontinuation of the study owing to an adverse event in the treatment group (4.2 %) compared with the placebo group (1.6 %). Each of the dosing regimens of lumacaftor seemed to have similar efficacy, with the exception of pulmonary exacerbation outcomes, which were more favorable in the 400 mg twice-daily lumacaftor group. The magnitude of improvement in FEV_1_ was less substantial in these studies than in the large Gly551Asp ivacaftor trial, but is comparable to the improvements demonstrated with other interventions in cystic fibrosis [6, 25, 71].

It is important to note that *in vitro* studies published in 2014 have suggested that treatment with ivacaftor over a longer period actually destabilizes the Phe508del-CFTR protein that is corrected by treatment with lumacaftor [72, 73]. These observations may partly explain the modest clinical improvements seen in the phase III TRAFFIC and TRANSPORT RCTs and highlight the importance of longer-term studies and awareness of drug–drug interactions involving novel compounds in chronic medical conditions for which polypharmacy is already the norm [8].

#### VX-661 and ivacaftor

VX-661 is another orally administered CFTR corrector small-molecule drug [74]. *In vitro*, VX-661 has been reported to improve trafficking and processing of Phe508del-CFTR and to have an additive effect when administered with ivacaftor on chloride transport compared with ivacaftor alone in cells heterozygous for Phe508del/Gly551Asp *CFTR* mutations [75]. The preliminary results of a complex phase II study investigating the safety and tolerability of VX-661 monotherapy and in combination with ivacaftor in patients who are homozygous for Phe508del and heterozygous for Phe508del/Gly551Asp *CFTR* mutations were presented at the 2014 North American Cystic Fibrosis Conference [75]. The primary outcome was safety and change in sweat chloride levels after 28 days of treatment. The study was blinded, randomized and placebo-controlled, and involved different dosing regimens.

The preliminary results suggest a modest decrease in sweat chloride levels with VX-661/ivacaftor combination treatment in Phe508del homozygous patients, with a dose-dependent increase in FEV_1_ in the treatment group compared with the placebo group that was statistically significant in the two highest dose groups (+4.8 percentage points predicted, *P* = 0.01). In Phe508del/Gly551Asp heterozygote participants, combination treatment was associated with non-statistically significant numerical decreases in sweat chloride levels, along with a statistically significant increase in FEV_1_ (+4.6 percentage points, *P* = 0.012).

## Future drug development of CFTR-targeted therapies

Our understanding of the precise mechanism of action of CFTR potentiators and correctors is increasing but remains incomplete. This challenge partly reflects the biological complexity of CFTR as a protein and the intricacy of its interactions. The CFTR correctors lumacaftor and VX-661 have been demonstrated to stabilize folding defects between different domains of Phe508del-CFTR [76]. It is possible that future CFTR correctors will further stabilize Phe508del-CFTR synergistically by targeting other protein domains, thus allowing greater trafficking of CFTR to the apical surface of cells [77, 78]. Increased knowledge of the mechanisms of action of CFTR-modulating drugs, along with the combination of more advanced and tractable experimental models, such as intestinal organoids [79], primary airway epithelial cell cultures [80, 81] and the application of genome editing technologies to stem cells [82], with high-throughput screening technologies, are likely to yield other small-molecule drugs for future treatment of CFTR defects.

As mentioned earlier, almost 2000 individual *CFTR* mutations have been identified, and particular challenges exist around developing therapeutic strategies for *CFTR* mutations that are individually very rare. These apply to both basic drug discovery, where combined efforts to generate biobanks of primary cells and tissues or use of genetic techniques in cells to allow research in specific mutations are likely to be necessary, and in demonstrating clinical efficacy. In the case of individually rare mutations, ‘*n*-of-1’ level *in vivo* evidence of efficacy generated by crossover studies may be important in assessing and developing future CFTR modulation strategies and facilitating access to new drugs for patients [83].

The data from clinical trials of lumacaftor/ivacaftor and VX-661/ivacaftor combination therapies support the concept of the corrector/potentiator pharmacological approach in patients who are homozygous for the Phe508del *CFTR* mutation, although further optimization of CFTR-potentiating drugs is likely to be required to yield maximal clinical benefits from combination therapy with ivacaftor. There may also be the potential to use combination therapy in patients who are heterozygous for Phe508del/Gly551Asp *CFTR* mutations to build further on the benefits associated with ivacaftor monotherapy.

## Impact of targeted therapies on care

The introduction of ivacaftor as a mutation-specific treatment that addresses the fundamental defect in people with the Gly551Asp mutation has been hugely exciting for patients with cystic fibrosis and clinicians alike. It represents one of the most powerful examples of precision medicine to date. Improvements in lung function demonstrated in RCTs, in conjunction with significant reductions in sweat chloride levels, are without precedent in cystic fibrosis and appear to be disease-modifying. The potential of small-molecule drugs used in combination to modulate CFTR function in other, much more prevalent mutations in the cystic fibrosis population, most notably Phe508del, is real, although the exact role and ‘real-life’ clinical efficacy of this approach are still being debated and optimized. The prospect of introducing CFTR-modulating therapies at a very early stage of life, potentially in utero, and thereby limiting organ damage and preserving function is also conceptually very attractive.

Optimism is appropriate but must be tempered against experience and the realities of drug development. Importantly, the longer-term benefits and effects of CFTR-modulating treatment are yet to be completely elucidated. Additionally, ivacaftor is an extremely expensive drug that costs in the region of US$300,000 annually per patient. Cost–benefit analyses are fraught with difficulty, because treatment would be lifelong by definition, but may also be disease modifying and be associated with reductions in other treatment costs, as well as potentially having wider societal benefits from increasing the health of people with cystic fibrosis [84]. Ultimately, a new model of drug development may evolve for orphan conditions such as cystic fibrosis, but in the meantime such targeted treatments are more expensive than previously accepted thresholds for quality-adjusted life years [85]. The less substantial improvements in lung function demonstrated in the TRAFFIC and TRANSPORT studies and approval of ivacaftor/lumacaftor in 2015 by the FDA in the United States are likely to generate further discussions about the cost–benefit ratio associated with treatment [7].

Cystic fibrosis is a complex disease and it is well recognized that although the principals underlying the genetic and functional defects in CFTR have been identified, two individuals with the same *CFTR* genotype may follow a different natural history and trajectory of their lung disease. The reason for this is undoubtedly multifactorial, including environmental, microbiological and socioeconomic factors, as well as adherence with treatment, with modifier genes also playing a role [86–89]. This heterogeneity adds a further challenge in evaluating responses to new therapeutic interventions and may explain differences in responses between individual patients. In this regard, precision medicine arguably remains a relative term in this area until our understanding increases. It is also likely that heterogeneity between individuals extends to the pharmacokinetics of CFTR-modulating drugs and biomarker responses.

## Conclusions

We have entered a new era of precision medicine in cystic fibrosis. Such precise treatments offer huge potential to target the underlying defects in specific CFTR mutations and alter the disease process, and could be life-changing. However, major challenges lie ahead to demonstrate longer-term benefits of these drugs, to develop compounds that target the most common classes of CFTR mutations, and to establish financially sustainable models of drug development and delivery [90].

## Abbreviations

ASL: Airway surface liquid
BMI: Body mass index
CBF: Ciliary beat frequency
CF: Cystic fibrosis
CFQ-R: Revised cystic fibrosis questionnaire
EC_50_: half-maximum effective concentration
FDA: Food and Drug Administration
FEV_1_: Baseline forced expiratory volume in 1 second
HBE: Human bronchial epithelial cell
kb: Kilobases
LCI: lung clearance index
PTC: Premature termination codon
RCT: Randomized controlled trial

## References

[1] Chirieac LR (2012). Emerging targeted therapies in cancer. Arch Pathol Lab Med.

[2] Slamon DJ, Leyland-Jones B, Shak S, Fuchs H, Paton V, Bajamonde A (2001). Use of chemotherapy plus a monoclonal antibody against HER2 for metastatic breast cancer that overexpresses HER2. N Engl J Med.

[3] Collins FS, Varmus H (2015). A new initiative on precision medicine. N Engl J Med.

[4] Hall IP (2013). Stratified medicine: drugs meet genetics. Eur Respir Rev.

[5] Riordan JR, Rommens JM, Kerem B, Alon N, Rozmahel R, Grzelczak Z (1989). Identification of the cystic fibrosis gene: cloning and characterization of complementary DNA. Science.

[6] Ramsey BW, Davies J, McElvaney NG, Tullis E, Bell SC, Drevinek P (2011). A CFTR potentiator in patients with cystic fibrosis and the G551D mutation. N Engl J Med.

[7] FDA approves new treatment for cystic fibrosis. http://www.fda.gov/newsevents/newsroom/pressannouncements/ucm453565.htm.

[8] Wainwright CE, Elborn JS, Ramsey BW, Marigowda G, Huang X, Cipolli M (2015). Lumacaftor–ivacaftor in patients with cystic fibrosis homozygous for Phe508del CFTR. N Engl J Med.

[9] O’Sullivan BP, Freedman SD (2009). Cystic fibrosis. Lancet.

[10] Corvol H, Thompson KE, Tabary O, le Rouzic P, Guillot L (2015). Translating the genetics of cystic fibrosis to personalized medicine. Transl Res.

[11] Bompadre SG, Sohma Y, Li M, Hwang TC (2007). G551D and G1349D, two CF-associated mutations in the signature sequences of CFTR, exhibit distinct gating defects. J Gen Physiol.

[12] Cheng SH, Gregory RJ, Marshall J, Paul S, Souza DW, White GA (1990). Defective intracellular transport and processing of CFTR is the molecular basis of most cystic fibrosis. Cell.

[13] Lukacs GL, Chang XB, Bear C, Kartner N, Mohamed A, Riordan JR (1993). The ΔF508 mutation decreases the stability of cystic fibrosis transmembrane conductance regulator in the plasma membrane. Determination of functional half-lives on transfected cells. J Biol Chem.

[14] Sheppard DN, Welsh MJ (1999). Structure and function of the CFTR chloride channel. Physiol Rev.

[15] Welsh MJ, Smith AE (1993). Molecular mechanisms of CFTR chloride channel dysfunction in cystic fibrosis. Cell.

[16] Dalemans W, Barbry P, Champigny G, Jallat S, Dott K, Dreyer D (1991). Altered chloride ion channel kinetics associated with the ΔF508 cystic fibrosis mutation. Nature.

[17] Matsui H, Grubb BR, Tarran R, Randell SH, Gatzy JT, Davis CW (1998). Evidence for periciliary liquid layer depletion, not abnormal ion composition, in the pathogenesis of cystic fibrosis airways disease. Cell.

[18] Button B, Cai LH, Ehre C, Kesimer M, Hill DB, Sheehan JK (2012). A periciliary brush promotes the lung health by separating the mucus layer from airway epithelia. Science.

[19] Pezzulo AA, Tang XX, Hoegger MJ, Alaiwa MH, Ramachandran S, Moninger TO (2012). Reduced airway surface pH impairs bacterial killing in the porcine cystic fibrosis lung. Nature.

[20] Cohen-Cymberknoh M, Kerem E, Ferkol T, Elizur A (2013). Airway inflammation in cystic fibrosis: molecular mechanisms and clinical implications. Thorax.

[21] Brodlie M, McKean MC, Johnson GE, Gray J, Fisher AJ, Corris PA (2010). Ceramide is increased in the lower airway epithelium of people with advanced cystic fibrosis lung disease. Am J Resp Crit Care Med.

[22] Teichgraber V, Ulrich M, Endlich N, Riethmuller J, Wilker B, De Oliveira-Munding CC (2008). Ceramide accumulation mediates inflammation, cell death and infection susceptibility in cystic fibrosis. Nat Med.

[23] Somaraju UR, Solis-Moya A (2014). Pancreatic enzyme replacement therapy for people with cystic fibrosis. Cochrane Database Syst Rev.

[24] Warnock L, Gates A, van der Schans CP (2013). Chest physiotherapy compared to no chest physiotherapy for cystic fibrosis. Cochrane Database Syst Rev.

[25] Jones AP, Wallis C (2010). Dornase alfa for cystic fibrosis. Cochrane Database Syst Rev.

[26] Elkins MR, Robinson M, Rose BR, Harbour C, Moriarty CP, Marks GB (2006). A controlled trial of long-term inhaled hypertonic saline in patients with cystic fibrosis. N Engl J Med.

[27] Smyth AR, Walters S (2014). Prophylactic anti-staphylococcal antibiotics for cystic fibrosis. Cochrane Database Syst Rev.

[28] Mahadeva R, Webb K, Westerbeek RC, Carroll NR, Dodd ME, Bilton D (1998). Clinical outcome in relation to care in centres specialising in cystic fibrosis: cross sectional study. BMJ.

[29] Dodge JA, Lewis PA, Stanton M, Wilsher J (2007). Cystic fibrosis mortality and survival in the UK: 1947–2003. Eur Respir J.

[30] De Boeck K, Wilschanski M, Castellani C, Taylor C, Cuppens H, Dodge J (2006). Cystic fibrosis: terminology and diagnostic algorithms. Thorax.

[31] Modi AC, Quittner AL (2006). Barriers to treatment adherence for children with cystic fibrosis and asthma: what gets in the way?. J Pediatr Psychol.

[32] McKone EF, Emerson SS, Edwards KL, Aitken ML (2003). Effect of genotype on phenotype and mortality in cystic fibrosis: a retrospective cohort study. Lancet.

[33] Armstrong DK, Cunningham S, Davies JC, Alton EW (2014). Gene therapy in cystic fibrosis. Arch Dis Child.

[34] Alton EWFW, Armstrong DK, Ashby D, Bayfield KJ, Bilton D, Bloomfield EV (2015). Repeated nebulisation of non-viral CFTR gene therapy in patients with cystic fibrosis: a randomised, double-blind, placebo-controlled, phase 2b trial. Lancet Respir Med.

[35] Schwank G, Koo BK, Sasselli V, Dekkers JF, Heo I, Demircan T (2013). Functional repair of CFTR by CRISPR/Cas9 in intestinal stem cell organoids of cystic fibrosis patients. Cell Stem Cell.

[36] Van Goor F, Hadida S, Grootenhuis PD, Burton B, Cao D, Neuberger T (2009). Rescue of CF airway epithelial cell function *in vitro* by a CFTR potentiator, VX-770. Proc Natl Acad Sci U S A.

[37] Cai ZW, Liu J, Li HY, Sheppard DN (2011). Targeting F508del-CFTR to develop rational new therapies for cystic fibrosis. Acta Pharmacol Sin.

[38] Farinha CM, Matos P, Amaral MD (2013). Control of cystic fibrosis transmembrane conductance regulator membrane trafficking: not just from the endoplasmic reticulum to the Golgi. FEBS J.

[39] Denning GM, Anderson MP, Amara JF, Marshall J, Smith AE, Welsh MJ (1992). Processing of mutant cystic fibrosis transmembrane conductance regulator is temperature-sensitive. Nature.

[40] Van Goor F, Hadida S, Grootenhuis PD, Burton B, Stack JH, Straley KS (2011). Correction of the F508del-CFTR protein processing defect *in vitro* by the investigational drug VX-809. Proc Natl Acad Sci U S A.

[41] Ledford H (2012). Drug bests cystic-fibrosis mutation. Nature.

[42] Accurso FJ, Rowe SM, Clancy JP, Boyle MP, Dunitz JM, Durie PR (2010). Effect of VX-770 in persons with cystic fibrosis and the G551D-CFTR mutation. N Engl J Med.

[43] Davies JC, Wainwright CE, Canny GJ, Chilvers MA, Howenstine MS, Munck A (2013). Efficacy and safety of ivacaftor in patients aged 6 to 11 years with cystic fibrosis with a G551D mutation. Am J Resp Crit Care Med.

[44] Davies J, Sheridan H, Bell N, Cunningham S, Davis SD, Elborn JS (2013). Assessment of clinical response to ivacaftor with lung clearance index in cystic fibrosis patients with a G551D-CFTR mutation and preserved spirometry: a randomised controlled trial. Lancet Respir Med.

[45] Barry PJ, Plant BJ, Nair A, Bicknell S, Simmonds NJ, Bell NJ (2014). Effects of ivacaftor in patients with cystic fibrosis who carry the G551D mutation and have severe lung disease. Chest.

[46] Sermet-Gaudelus I (2013). Ivacaftor treatment in patients with cystic fibrosis and the G551D-CFTR mutation. Eur Resp Rev.

[47] Yu H, Burton B, Huang CJ, Worley J, Cao D, Johnson JP (2012). Ivacaftor potentiation of multiple CFTR channels with gating mutations. J Cyst Fibros.

[48] Van Goor F, Yu H, Burton B, Hoffman BJ (2014). Effect of ivacaftor on CFTR forms with missense mutations associated with defects in protein processing or function. J Cyst Fibros.

[49] de Nooijer RA, Nobel JM, Arets HG, Bot AG, van Berkhout FT, de Rijke YB (2011). Assessment of CFTR function in homozygous R117H-7 T subjects. J Cyst Fibros.

[50] Van Goor F, Straley KS, Cao D, Gonzalez J, Hadida S, Hazlewood A (2006). Rescue of ΔF508-CFTR trafficking and gating in human cystic fibrosis airway primary cultures by small molecules. Am J Physiol.

[51] De Boeck K, Munck A, Walker S, Faro A, Hiatt P, Gilmartin G (2014). Efficacy and safety of ivacaftor in patients with cystic fibrosis and a non-G551D gating mutation. J Cyst Fibros.

[52] Thauvin-Robinet C, Munck A, Huet F, Genin E, Bellis G, Gautier E (2009). The very low penetrance of cystic fibrosis for the R117H mutation: a reappraisal for genetic counselling and newborn screening. J Med Genet.

[53] Moss RB, Flume PA, Elborn JS, Cooke J, Rowe SM, McColl SA, et al. Efficacy and safety of ivacaftor treatment in subjects with cystic fibrosis who have an R117H-CFTR mutation. Lancet Respir Med. 2015. In press. 10.1016/S2213-2600(15)00201-5 PMC4641035 26070913

[54] Flume PA, Liou TG, Borowitz DS, Li H, Yen K, Ordonez CL, Geller DE (2012). Ivacaftor in subjects with cystic fibrosis who are homozygous for the F508del-CFTR mutation. Chest.

[55] Borthwick LA, Botha P, Verdon B, Brodlie MJ, Gardner A, Bourn D (2011). Is CFTR-delF508 really absent from the apical membrane of the airway epithelium?. PLoS One.

[56] McKone EF, Borowitz D, Drevinek P, Griese M, Konstan MW, Wainwright C (2014). Long-term safety and efficacy of ivacaftor in patients with cystic fibrosis who have the Gly551Asp-CFTR mutation: a phase 3, open-label extension study (PERSIST). Lancet Respir Med.

[57] Rowe SM, Heltshe SL, Gonska T, Donaldson SH, Borowitz D, Gelfond D (2014). Clinical mechanism of the cystic fibrosis transmembrane conductance regulator potentiator ivacaftor in G551D-mediated cystic fibrosis. Am J Resp Crit Care Med.

[58] Molloy K, McElvaney NG (2014). Ivacaftor: from bench to bedside…and back again. Am J Resp Crit Care Med.

[59] Sermet-Gaudelus I, Boeck KD, Casimir GJ, Vermeulen F, Leal T, Mogenet A (2010). Ataluren (PTC124) induces cystic fibrosis transmembrane conductance regulator protein expression and activity in children with nonsense mutation cystic fibrosis. Am J Resp Crit Care Med.

[60] Welch EM, Barton ER, Zhuo J, Tomizawa Y, Friesen WJ, Trifillis P (2007). PTC124 targets genetic disorders caused by nonsense mutations. Nature.

[61] Thursfield RM, Davies JC (2012). Cystic fibrosis: therapies targeting specific gene defects. Paediatr Respir Rev.

[62] McElroy SP, Nomura T, Torrie LS, Warbrick E, Gartner U, Wood G (2013). A lack of premature termination codon read-through efficacy of PTC124 (Ataluren) in a diverse array of reporter assays. PLoS Biol.

[63] Bushby K, Finkel R, Wong B, Barohn R, Campbell C, Comi GP (2014). Ataluren treatment of patients with nonsense mutation dystrophinopathy. Muscle Nerve.

[64] Kerem E, Hirawat S, Armoni S, Yaakov Y, Shoseyov D, Cohen M (2008). Effectiveness of PTC124 treatment of cystic fibrosis caused by nonsense mutations: a prospective phase II trial. Lancet.

[65] Wilschanski M, Miller LL, Shoseyov D, Blau H, Rivlin J, Aviram M (2011). Chronic ataluren (PTC124) treatment of nonsense mutation cystic fibrosis. Eur Respir J.

[66] Kerem E, Konstan MW, De Boeck K, Accurso FJ, Sermet-Gaudelus I, Wilschanski M (2014). Ataluren for the treatment of nonsense-mutation cystic fibrosis: a randomised, double-blind, placebo-controlled phase 3 trial. Lancet Respir Med.

[67] Study of Ataluren in Nonsense Mutation Cystic Fibrosis (ACT CF). https://clinicaltrials.gov/ct2/show/NCT02139306.

[68] Clancy JP, Rowe SM, Accurso FJ, Aitken ML, Amin RS, Ashlock MA (2012). Results of a phase IIa study of VX-809, an investigational CFTR corrector compound, in subjects with cystic fibrosis homozygous for the F508del-CFTR mutation. Thorax.

[69] Lane MA, Doe SJ (2014). A new era in the treatment of cystic fibrosis. Clin Med.

[70] Boyle MP, Bell SC, Konstan MW, McColley SA, Rowe SM, Rietschel E (2014). A CFTR corrector (lumacaftor) and a CFTR potentiator (ivacaftor) for treatment of patients with cystic fibrosis who have a phe508del CFTR mutation: a phase 2 randomised controlled trial. Lancet Respir Med.

[71] Mogayzel PJ, Naureckas ET, Robinson KA, Mueller G, Hadjiliadis D, Hoag JB (2013). Cystic fibrosis pulmonary guidelines. Chronic medications for maintenance of lung health. Am J Resp Crit Care Med.

[72] Cholon DM, Quinney NL, Fulcher ML, Esther CR, Das J, Dokholyan NV (2014). Potentiator ivacaftor abrogates pharmacological correction of ΔF508 CFTR in cystic fibrosis. Sci Transl Med.

[73] Veit G, Avramescu RG, Perdomo D, Phuan PW, Bagdany M, Apaja PM (2014). Some gating potentiators, including VX-770, diminish ΔF508-CFTR functional expression. Sci Transl Med.

[74] Pettit RS, Fellner C (2014). CFTR Modulators for the treatment of cystic fibrosis. Proc Natl Acad Sci U S A.

[75] Pilewski JM, Donaldson SH, Cooke J, Lekstrom-Himes J (2014). Phase 2 studies reveal additive effects of VX-661, an investigational CFTR corrector and ivacaftor, a CFTR potentiator, in patients with CF who carry the d508-CFTR mutation. Ped Pulmonol.

[76] Ren HY, Grove DE, De La Rosa O, Houck SA, Sopha P, Van Goor F (2013). VX-809 corrects folding defects in cystic fibrosis transmembrane conductance regulator protein through action on membrane-spanning domain 1. Mol Biol Cell.

[77] Okiyoneda T, Veit G, Dekkers JF, Bagdany M, Soya N, Xu H (2013). Mechanism-based corrector combination restores ΔF508-CFTR folding and function. Nat Chem Biol.

[78] Phuan PW, Veit G, Tan J, Roldan A, Finkbeiner WE, Lukacs GL (2014). Synergy-based small-molecule screen using a human lung epithelial cell line yields ΔF508-CFTR correctors that augment VX-809 maximal efficacy. Mol Pharmacol.

[79] Dekkers JF, Wiegerinck CL, de Jonge HR, Bronsveld I, Janssens HM, de Winter-de Groot KM (2013). A functional CFTR assay using primary cystic fibrosis intestinal organoids. Nat Med.

[80] Brodlie M, McKean MC, Johnson GE, Perry JD, Nicholson A, Verdon B (2010). Primary bronchial epithelial cell culture from explanted cystic fibrosis lungs. Exp Lung Res.

[81] de Courcey F, Zholos AV, Atherton-Watson H, Williams MT, Canning P, Danahay HL (2012). Development of primary human nasal epithelial cell cultures for the study of cystic fibrosis pathophysiology. Am J Physiol.

[82] Crane AM, Kramer P, Bui JH, Chung WJ, Li XS, Gonzalez-Garay ML (2015). Targeted correction and restored function of the CFTR gene in cystic fibrosis induced pluripotent stem cells. Stem Cell Rep.

[83] Lillie EO, Patay B, Diamant J, Issell B, Topol EJ, Schork NJ (2011). The n-of-1 clinical trial: the ultimate strategy for individualizing medicine?. Per Med.

[84] Whiting P, Al M, Burgers L, Westwood M, Ryder S, Hoogendoorn M (2014). Ivacaftor for the treatment of patients with cystic fibrosis and the G551D mutation: a systematic review and cost-effectiveness analysis. Health Technol Assess.

[85] Balfour-Lynn IM (2014). Personalised medicine in cystic fibrosis is unaffordable. Paediatr Respir Rev.

[86] Santis G, Osborne L, Knight RA, Hodson ME (1990). Linked marker haplotypes and the ΔF508 mutation in adults with mild pulmonary disease and cystic fibrosis. Lancet.

[87] Kerem E, Corey M, Gold R, Levison H (1990). Pulmonary function and clinical course in patients with cystic fibrosis after pulmonary colonization with Pseudomonas aeruginosa. J Pediatr.

[88] Barr HL, Britton J, Smyth AR, Fogarty AW (2011). Association between socioeconomic status, sex, and age at death from cystic fibrosis in England and Wales (1959 to 2008): cross sectional study. BMJ.

[89] Arkwright PD, Laurie S, Super M, Pravica V, Schwarz MJ, Webb AK (2000). TGF-β_1_ genotype and accelerated decline in lung function of patients with cystic fibrosis. Thorax.

[90] Cohen D, Raftery J (2014). Paying twice: questions over high cost of cystic fibrosis drug developed with charitable funding. BMJ.

